# *In Vitro* Assembly of Catalase*

**DOI:** 10.1074/jbc.M114.596148

**Published:** 2014-08-22

**Authors:** Michael Baureder, Elisabeth Barane, Lars Hederstedt

**Affiliations:** From the Microbiology Group, Department of Biology, Lund University, Sölvegatan 35, SE-22362 Lund, Sweden

**Keywords:** Bacterial Metabolism, Biosynthesis, Catalase, Enterococcus, Enzyme, Heme, Protein Assembly

## Abstract

Most aerobic organisms contain catalase, which functions to decompose hydrogen peroxide. Typical catalases are structurally complex homo-tetrameric enzymes with one heme prosthetic group buried in each subunit. It is not known how catalase in the cell is assembled from its constituents. The bacterium *Enterococcus faecalis* cannot synthesize heme but can acquire it from the environment to form a cytoplasmic catalase. We have in *E. faecalis* monitored production of the enzyme polypeptide (KatA) depending on the availability of heme and used our findings to devise a procedure for the purification of preparative amounts of *in vivo*-synthesized apocatalase. We show that fully active catalase can be obtained *in vitro* by incubating isolated apoprotein with hemin. We have characterized features of the assembly process and describe a temperature-trapped hemylated intermediate of the enzyme maturation process. Hemylation of apocatalase does not require auxiliary cell components, but rapid assembly of active enzyme seemingly is assisted in the cell. Our findings provide insight about catalase assembly and offer new experimental possibilities for detailed studies of this process.

## Teaser

**Background:** Catalase is a heme protein of intricate structural organization, and its assembly is not understood.

**Results:** Apocatalase in *E. faecalis* was found synthesized independent of heme, and procedures for its purification and hemylation were established.

**Conclusion:** Catalase can be assembled *in vitro*.

**Significance:** The reported findings open up for new *in vitro* experimental approaches to study heme protein biogenesis.

## Introduction

Canonical heme proteins contain one or more heme groups and have crucial functions in most organisms. *In vitro* systems for assembly of heme proteins are desired in research aiming at a full mechanistic understanding of heme protein biogenesis, including coordination of protein folding with hemylation (heme insertion). Such systems can also be instrumental for production of non-natural, “synthetic” proteins for biotechnical or basic research applications.

The structure and function of, for example, hemoglobin, myoglobin, and catalase are known in the details. However, it is not understood how these, and most other heme proteins, are assembled from the constituents, heme and polypeptide, in living cells.

One major obstacle that generally complicates, or even prevents, *in vitro* studies on how a heme protein is assembled is lack of heme-free apoprotein. Apoprotein can in some cases, for example hemoglobin and myoglobin, be obtained by dehemylation of isolated holoprotein (1). However, such a procedure often requires extreme pH values or organic solvents, and it can be difficult to remove all the heme and obtain apoprotein in a functional state. Most organisms contain heme and depend on it for growth. For this reason the apo-form of a heme protein cannot easily be produced in microbes or eukaryotic cells or synthesized by translation *in vitro* using cell extracts (which normally contain heme). In the work reported here we have devised a procedure for production of apoprotein in heme-less bacterial cells. Using special properties of the enteric lactic acid bacterium *Enterococcus faecalis* we demonstrate that apocatalase can be produced in that organism, purified, and used to *in vitro* assemble functional catalase. Previous attempts to assemble a typical heme catalase in cell extracts or from constituents *in vitro* have been reported unsuccessful (2–4).

In the presence of heme (protoheme IX) *E. faecalis* cells assemble two heme proteins, a membrane-bound cytochrome *bd* (5), and a cytoplasmic typical catalase (6). This bacterium lacks enzymes for tetrapyrrole synthesis and therefore cannot produce heme proteins unless heme is provided in the environment (7). It is not known how heme is transported into and inside the *E. faecalis* cell to be delivered to cytochrome and catalase apoproteins. Heme is a relatively large metallo-organic compound that is water-insoluble at neutral pH and toxic to cells due to the reactivity of the iron atom as ferrous heme in the presence of air generates reactive oxygen species.

*E. faecalis* catalase (KatA) belongs to clade 3 of typical small subunit (monofunctional) catalases (EC 1.11.1.6) and is very similar to mammalian catalase (8, 9). The crystal structure of the enzyme (10) reveals a homo-tetramer containing one protoheme IX group per 54-kDa subunit. The non-covalently bound heme is buried inside the enzyme, accessible only to small substrates through narrow channels. The quaternary structure of catalase shows interweaving of subunits, which involves passing polypeptide parts of one subunit through holes in another subunit. How this remarkable structure is assembled mechanistically is unsolved (2, 11, 12). The process presumably first comprises partial folding of the apoprotein followed by its hemylation and stepwise subunit oligomerization. The details and order of these events and requirement of auxiliary cell factors have not been established. Lack of apoprotein and an experimental system for *in vitro* assembly have restricted research on catalase biogenesis. In this work we present assembly of catalase from purified components and report findings that advance our knowledge about catalase biogenesis. Moreover, our results open up for new experimental approaches to unravel molecular details of the catalase maturation process.

## EXPERIMENTAL PROCEDURES

### 

#### 

##### Culture Media

*E. faecalis* strains used in this work are listed in Table 1. Cells were grown at 37 °C on Todd Hewitt agar or in tryptic soy broth (TSB^2^ without dextrose, Difco) supplemented with 1% glucose (TSBG). When required, 10 μg ml^−1^ chloramphenicol was added to the media. When indicated hemin (Sigma-Aldrich) was added to TSBG (a heme-free medium (6)) from a 10 mm stock solution in dimethyl sulfoxide (DMSO). The concentration of hemin in stock solutions was determined gravimetrically and confirmed by the pyridine hemochromogen method (13). *Escherichia coli* was grown at 37 °C in LB or on LB agar (LA) plates with or without 12.5 μg ml^−1^ chloramphenicol added.

##### Construction of Plasmids pLUMB29 and pLUMB31A

The *E. faecalis* OG1RF chromosomal *katA* gene was amplified by PCR using primers katA03 (5′-CGCTGAATGACCAATAAAAACG-3′) and katA04 (5′-CCCAACTAGCTTAGCAAACAAC-3′). The PCR product was cloned into vector pCR-Blunt II-TOPO, and *E. coli* TOP10 (Invitrogen) was transformed with the resulting plasmid, pLUMB29. Site-directed mutagenesis was done using the QuikChange site-directed mutagenesis kit (Stratagene) with primers katAC82A01 (5′-CTGCAGGAGAATTTGGTACTGTCCTAATCCAAGAC-3′) and katAC82A02 (5′-GTCTTGGATTAGGACAGTACCAAATTCTCCTGCAG-3′). The *katA82* mutation, underlined in the sequence of the primers, was confirmed by DNA sequence analysis. The mutant gene was transferred to the *E. coli*/*E. faecalis* shuttle vector pAM401, resulting in plasmid pLUMB31A, which was propagated in *E. coli* TOP10. Transformation of *E. faecalis* strain EMB4 with pLUMB31A was done as described by Dunny *et al.* (14).

##### Construction of pLUMB38

Plasmid pLUMB38, encoding KatA with a C-terminal StrepII-tag, was constructed by amplifying *E. faecalis katA* using primers C1 (5′-TGTGGATCCTGGTGGTGTAAACAG-3′) and katACStrep (5′-GACGAATTCTTATTTTTCGAACTGCGGGTGGCTCCAAGCGCTTGCTTGTTGCTTGAT-3′). The PCR product was cloned into pAM401 via the BamHI and EcoRI sites introduced by the primers. The obtained plasmid was propagated in *E. coli* TOP10 and used to transform *E. faecalis* strain ELF7.

##### Growth of E. faecalis and Analysis of Cell Extracts for KatA

Cells grown overnight on Todd Hewitt agar were used to inoculate 50 ml of TSBG to an OD_600_ of 0.05. The culture was incubated aerobically at 37 °C and 200 rpm until OD_600_ reached 1.0. Then it was diluted to an OD_600_ of 0.05 in two 5-liter baffled Erlenmeyer flasks each containing 500 ml of freshly prepared prewarmed medium, and incubation was continued. When OD_600_ reached 0.2 a sample was taken from each culture, and subsequently hemin was added to one flask and DMSO was added to the other. Incubation was continued, and additional samples were taken at several time points. For each sample 25–50 ml of the bacterial culture were transferred to a centrifuge tube containing 5–10 g of crushed ice. Cells were harvested by centrifugation at 8,000 × *g* and 4 °C for 10 min. The cell pellet was washed once in 10–20 ml of cold TES (50 mm Tris·HCl, pH 7.5, 5 mm EDTA, 50 mm NaCl) and finally stored at −20 °C.

Cell pellets were thawed and suspended in 50 mm KPO_4_, pH 8.0. The suspension was transferred to 2-ml screw-cap tubes containing 1.75 g of zirconia/silica beads (diameter = 0.1 mm). Cell lysis was done in a FastPrep instrument (MP Biomedicals) for 3 × 20 s at 6 m s^−1^. Debris and unbroken cells were removed by centrifugation for 30 min at 5,000 × *g* and 4 °C. The supernatants (cell lysate) were stored at −20 °C.

##### Total RNA Extraction and Quantitative RT-PCR

Two cultures of *E. faecalis* strain OG1RF were grown as described above. Hemin was added at a final concentration of 0.1 μm, samples of 12.5 ml were taken 10 min before and 15, 30, 60, and 120 min after hemin (or DMSO) addition, and the washed cell pellets were stored at −80 °C. Extraction of total RNA was done using the RNeasy mini kit (Qiagen) with on-column DNase treatment according to the manufacturer's instructions. For cell lysis the pellets were suspended in 1 ml of buffer RLT, transferred to screw-cap tubes containing 1.75 g of zirconia/silica beads (diameter = 0.1 mm), and run in a FastPrep instrument for 2 × 20 s at 6 m s^−1^. Unbroken cells and debris were removed by centrifugation for 10 s at 14,000 × *g*. The concentration of the isolated RNA was determined using a NanoDrop spectrophotometer, and RNA quality was confirmed on agarose gels. Synthesis of cDNA was done from 2 μg of RNA using Superscript III reverse transcriptase (Invitrogen) and random hexamers according to the manual. For each RNA preparation a control reaction lacking reverse transcriptase (−RT) was run to exclude DNA contamination.

For quantitative RT-PCR the cDNA was diluted 5-fold in 10 mm Tris·HCl, pH 8.0, and 2 μl were added to 23 μl of Maxima SYBR Green master mix (Fermentas) containing primers KatAF01 (5′-CGTCATATGCATGGTTTTGG-3′) and KatAR01 (5′-TCATCGTGCCAACTTCAATC-3′). The PCR reaction was run in triplicates in a Rotor-Gene 2072 instrument (Corbett Research). Comparative quantification of three biological replicates (cell growth and RNA extraction) was done using the Rotor-Gene 6.0 software.

##### Purification of Hexahistidyl-tagged Apo- and Holocatalase from E. faecalis Cells

Cells of strain ELF7/pLUF15 were used to inoculate 250 ml of TSBG containing chloramphenicol to an OD_600_ of 0.05. The culture was incubated aerobically at 37 °C and 200 rpm. When OD_600_ reached 0.5 the culture was diluted in 2 liters of prewarmed medium distributed over four 5-liter baffled Erlenmeyer flasks and incubation was continued. At OD_600_ = 1 the culture was transferred to three 1-liter centrifuge flasks each containing 200 g of crushed ice. Cells were harvested by centrifugation for 15 min at 8,000 × *g* and 4 °C. The cell pellet was washed once in 20 ml of cold TES or 50 mm KPO_4_, pH 8.0, solution and finally stored at −20 °C. For isolation of holocatalase, 8 μm hemin was included in the growth medium.

For lysis, cells from 2 liters of culture were suspended in a total volume of 15 ml of 50 mm KPO_4_, pH 8.0. The cells were disrupted as described under “Growth of E. faecalis and Analysis of Cell Extracts for KatA,” but after centrifugation the supernatants were pooled and centrifuged for 30 min at 50,000 × *g* and 4 °C to remove membranes. To the supernatant (cytoplasmic fraction) was added an equal volume of 50 mm KPO_4_ buffer, pH 8.0, containing 600 mm NaCl and 2 mm imidazole before it was passed through a 0.45-μm filter.

For KatA-His_6_ affinity chromatography a 1-ml HiTrap chelating HP column (GE Healthcare) connected to a pump operated at 1.5 ml min^−1^ was loaded with NiCl_2_ according to the manufacturer's instructions. The column was equilibrated with 5 ml of buffer A (50 mm KPO_4_, pH 8.0, 300 mm NaCl, 1 mm imidazole), 5 ml of buffer B (50 mm KPO_4_, pH 8.0, 300 mm NaCl, 30 mm imidazole), and 10 ml of buffer A. The sample was loaded onto the column and washed with 5 ml of buffer A and 5 ml of buffer B. During elution with 5 ml of 50 mm KPO_4_, pH 8.0, 300 mm NaCl, 250 mm imidazole fractions of 1 ml were collected. Fractions that contained KatA protein were identified by SDS-PAGE and subsequently pooled.

Buffer exchange to 50 mm KPO_4_, pH 8.0, and sample concentration were done using a centrifugal filtration device (Amicon Ultra, molecular weight cut-off 10 kDa). Dilution and concentration was repeated three times. Alternatively the buffer was changed to 50 mm KPO_4_, pH 8.0, using a PD MiniTrap G-25 column (GE Healthcare). Purified apo- and holocatalase were stored in the dark on an ice bath. Apocatalase was used within a week, and supplementation of the storage buffer with 1 mm PMSF protected the preparations against degradation. The KatA concentration in preparations of purified apocatalase was determined by quantitative immuno-blot. Band intensities (pixels) obtained from serial dilutions were compared with intensities from holocatalase of known quantities.

##### Production and Purification of StrepII-tagged Apo- and Holocatalase from E. faecalis Cells

For purification of StrepII-tagged catalase cells of strain ELF7/pLUMB38 were grown and disrupted as described for ELF7/pLUF15 (His-tagged catalase). For preparation of cytoplasmic fraction 100 mm Tris·HCl, pH 8.0, 150 mm NaCl was used instead of phosphate buffer. Gravity flow affinity chromatography was carried out on a 0.5-ml *Strep*-Tactin column (IBA) that was prepared according to the manufacturer's instructions. After loading the sample onto the column it was washed thrice with 0.5 ml of buffer W (100 mm Tris·HCl, pH 8.0, 150 mm NaCl) followed by elution of the KatA-*Strep* fusion protein with 6 × 0.25 ml of buffer E (100 mm Tris·HCl, pH 8.0, 150 mm NaCl, 2.5 mm desthiobiotin). The presence and purity of catalase protein in fractions collected during washing and elution was determined by SDS-PAGE and immuno-blot. For further analysis of the purified protein the buffer was changed to 50 mm KPO_4_, pH 8.0 using a PD MiniTrap G-25 column (GE Healthcare).

##### Analysis of KatA82

Cell lysates of *E. faecalis* strain EMB4/pLUMB31A grown overnight in hemin-supplemented TSBG were subjected to centrifugation for 30 min at 50,000 × *g* and 4 °C. The pellet containing insoluble material and membranes (2 μg ml^−1^ total protein) was suspended in 500 μl of 20 mm MOPS·HCl, pH 7.4, 0.4 m NaCl, 1 mm EDTA or this buffer containing 1% SDS, 1% *n*-dodecyl β-d-maltoside, 1% Triton X-100, or 8 m urea, respectively. After incubation for 1 h at room temperature a 20-μl aliquot was added to SDS-PAGE sample buffer (= total membrane fraction). The remaining suspension was centrifuged for 30 min at 50,000 × *g* and 4 °C to pellet unsolubilized material, and a 20-μl aliquot from the top part of the supernatant was added to SDS-PAGE sample buffer (= solubilized proteins). KatA in samples was determined by immuno-blot.

##### Stability Tests of KatA Polypeptide

Cell lysates (12.5 μg of total protein) of strain OG1RF grown in heme-free medium to late exponential growth phase were mixed with an equal amount of lysate from early exponential growth phase cells in a total volume of 50 μl of 50 mm KPO_4_, pH 8.0. The mixture was kept on ice, and a 20-μl aliquot was transferred to a tube containing 20 μl of 2× SDS-PAGE sample buffer. The remaining mixture was incubated at 37 °C, and aliquots were removed similarly after 15, 30, 60, and 120 min. KatA polypeptide in the samples was determined by immuno-blot.

To test protection of KatA by protease inhibitors 1 mm PMSF and/or 1 mm EDTA, or 1× Complete mini EDTA-free protease inhibitor cocktail (Roche Applied Science), were added to lysate from late exponential growth phase cells. The mixtures were incubated for 10 min on ice before proceeding as described here above.

##### Gel Electrophoresis and Immuno-blot

Proteins were separated under denaturing conditions using the NuPAGE system (Invitrogen) with 10% Bis-Tris gels and SDS MOPS running buffer. Proteins were transferred to a PVDF membrane (Millipore) by electroblotting in 20 mm Tris and 150 mm glycine in 20% (v/v) methanol, and KatA antigen was detected using rabbit anti-KatA antiserum (6) and horseradish peroxidase-coupled anti-rabbit antibodies (GE Healthcare). The SuperSignal West Pico kit (Pierce) and a Kodak Image station or ChemiDoc^TM^ MP imaging system (Bio-Rad) were used for signal detection.

##### Microtiter Plate Catalase Assay

Ten μl of purified holo- or apocatalase were diluted in 8 μl of 50 mm KPO_4_, pH 8.0, and mixed with 2 μl of a hemin stock solution in DMSO (0.1–10 μm) per well of a 96-well microtiter plate. Samples were incubated at 37 °C for 20 min. Zymogram staining reagent (15) was prepared by mixing 2 ml of 80 mg ml^−1^ dopamine in 0.2 m KPO_4_, pH 8.0, 2 ml of 40 mg ml^−1^ *p*-phenylenediamine in 0.2 m KPO_4_, pH 8.0, 2 ml of 12% hydrogen peroxide, and 2 ml of DMSO. The reagent was diluted 10-fold in 50 mm KPO_4_, pH 8.0, and 200 μl were added to each well of the plate. Catalase activity was detected by the development of purple color.

##### Gel Permeation Chromatography

Purified catalase protein was analyzed by chromatography on a Superose 6 10/300 GL column connected to an ÄKTA Avant chromatography system (GE Healthcare). The column was equilibrated in 50 mm KPO_4_, pH 8.0, 150 mm NaCl and run at 20 °C. One or three hundred μl of protein sample were injected and eluted at a flow rate of 0.5 ml min^−1^.

##### Catalase Activity Measurement

Sample (1–30 μl) was added to a cuvette containing 0.1% hydrogen peroxide in 1 ml of 50 mm KPO_4_, pH 7.0. The rate of hydrogen peroxide decomposition at 37 °C was recorded as the decrease in absorption at 240 nm, and activity was calculated based on the initial rate (up to 20 s). One unit was defined as the decomposition of 1 μmol of hydrogen peroxide/min.

##### Miscellaneous Techniques

Protein concentrations of cell lysates were determined using the BCA protein assay (Pierce) with serum albumin as reference protein. Heme concentrations were determined by the pyridine hemochromogen method (13). Spectra were recorded at room temperature using an upgraded (OLIS Instruments) Aminco DW-2 spectrophotometer and a slit width of 1 nm.

## RESULTS

### 

#### 

##### katA Transcription in E. faecalis Is Independent of Heme

*E. faecalis* cells take up heme from the growth medium to form active catalase (7, 16). In previous work it was found that the presence of KatA polypeptide in aerobic stationary growth phase cells is dependent on heme in the growth medium (6). This suggested that heme promotes transcription of *katA*, translation of *katA* mRNA, or stability of KatA polypeptide.

To determine whether the heme-dependent KatA concentration difference is reflected in mRNA concentrations we analyzed cellular *katA* mRNA using quantitative RT-PCR. *E. faecalis* strain OG1RF (Table 1) grown in hemin-supplemented and heme-free TSBG medium as described under “Experimental Procedures” showed no difference in abundance of *katA* mRNA. This indicated that the *katA* gene is constitutively expressed under the growth conditions used; *i.e.* it is not induced by the presence of heme in the growth medium.

**TABLE 1:** ***E. faecalis* strains and plasmids used in this work** Cm^r^, Kan^r^, and Tet^r^ indicate resistance to chloramphenicol, kanamycin, and tetracycline, respectively.

Strain or plasmid	Relevant characteristics	Source/reference
OG1RF	Parental strain	22
ELF7	*katA::tet*; Tet^r^	23
EMB4	*katA82 cydC::Tn;* Tet^r^	17
pAM401	*E. coli*/*E. faecalis* shuttle vector; Tet^r^, Cm^r^	22
pLUF15	*katA*(His)_6_ in pAM401; Cm^r^	6
pLUMB29	*katA* in pCR-Blunt II-TOPO; Kan^r^	This work
pLUMB31A	*katA82* in pAM401; Cm^r^	This work
pLUMB38	*katA*(*Strep*-tag II) in pAM401; Cm^r^	This work

##### Synthesis of KatA Polypeptide in E. faecalis Does Not Depend on Heme

**FIGURE 1.**
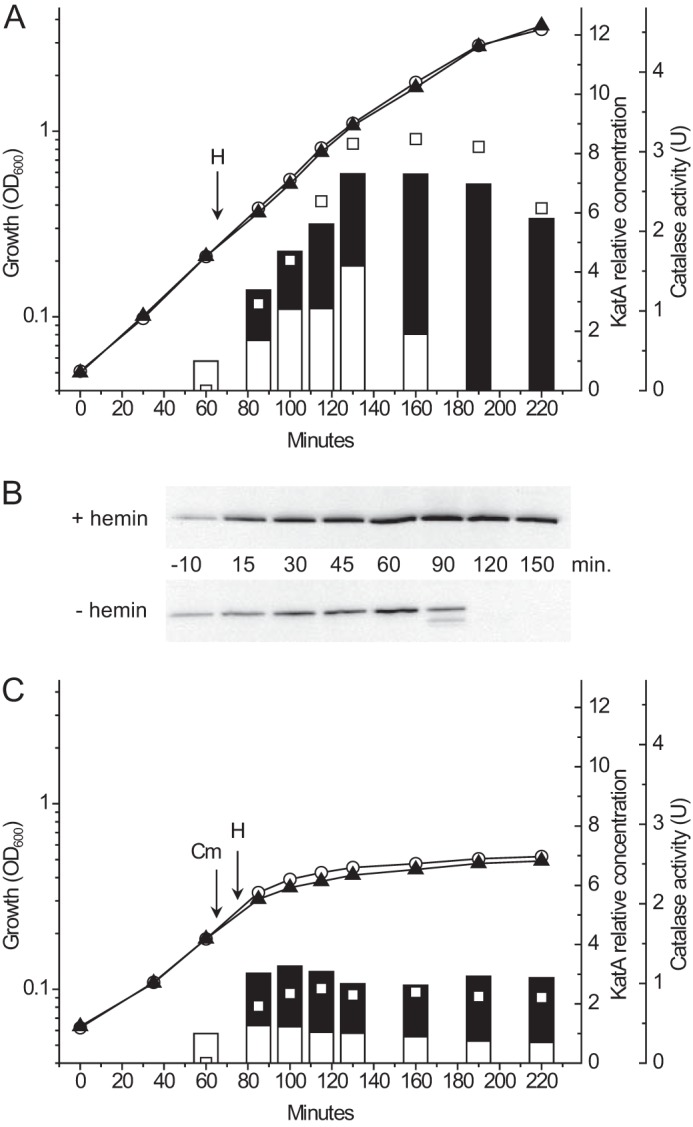
**The cellular level of catalase polypeptide in *E. faecalis* is dependent on availability of heme and growth phase.** *A*, cells of *E. faecalis* OG1RF were grown in TSBG. Hemin (*H*) (8 μm, *filled triangles*) or DMSO (*open circles*) was added in early exponential growth phase (*arrow*). Catalase (KatA) polypeptide in cell lysates was determined by immuno-blot (*panel B*), and the amount is in the form of a bar presented relative to that in the first collected sample. Catalase activity is shown for the hemin-supplemented culture (*open squares*). *OD*, optical density; *U*, units. *C*, the experiment shown in *panel A* repeated except that protein synthesis was inhibited by the addition of chloramphenicol (*Cm*) prior to hemin (*H*).

To analyze the effect of heme on KatA polypeptide synthesis and stability, strain OG1RF was grown in the absence of heme until early exponential growth phase (OD_600_ = 0.2). At that point 8 μm hemin (final concentration) was added to one part of the culture, and a corresponding volume of solvent (DMSO) was added to the other part of the culture and incubation was continued. Samples were taken from both cultures before and at several time points after the addition of hemin (or DMSO). Growth under the conditions used was essentially unaffected by the presence of hemin (Fig. 1*A*). KatA polypeptide, as determined by immuno-blot using polyclonal antiserum, was found in cells of both cultures. In the culture without heme, the cellular concentration of KatA increased during exponential growth and then declined at early stationary growth phase until, in samples taken at 120 and 150 min, no KatA polypeptide could be found (17) (Fig. 1, *A* and *B*). A larger increase in the cellular amount of KatA was observed in the hemin-supplemented culture and, in contrast to the culture without hemin, KatA polypeptide remained in the cells also at early stationary growth phase. Catalase activity in the hemin-supplemented cells correlated with the KatA polypeptide concentration (Fig. 1*A*), which confirmed the presence of holocatalase. As expected no catalase activity was detected in samples taken prior to hemin addition.

The results showed that KatA polypeptide is synthesized in the absence of heme but degraded in late exponential growth phase. Proteolytic degradation was observed by appearance of fragments of KatA polypeptide detectable by immuno-blot of samples taken at 90 min from the culture grown without hemin (Fig. 1*B*). These degradation products were not found in samples from the culture supplemented with hemin.

The hemin concentration in the culture medium required to saturate biogenesis of holocatalase was analyzed by adding 0.1, 1, and 8 μm hemin to cultures at mid-exponential growth phase. The amount of KatA in cell lysates was determined by immuno-blot before and at several time points after hemin addition. No significant difference in KatA concentration could be seen at ≥1 μm hemin. With 0.1 μm hemin the final yield of KatA was reduced to about half, showing that formation of holocatalase in the cells is limited at that heme concentration (data not shown).

To analyze KatA maturation *in vivo* more closely, the experiment shown in Fig. 1*A* was repeated but protein synthesis was blocked by the addition of 100 μg ml^−1^ chloramphenicol to the cultures 10 min before hemin or DMSO was added. Under these conditions catalase activity increased rapidly after hemin addition and then did not increase further (Fig. 1*C*). This is in accordance with original observations reported by Pugh and Knowles (18) who showed that catalase activity in *E. faecalis* can be obtained almost instantaneously after the addition of hemin to aerobically grown cells and is as expected if apocatalase is present in the cells before heme is added. Cells of the hemin-deficient culture contained an essentially constant amount of KatA after protein synthesis was blocked by the addition of chloramphenicol, indicating no degradation of KatA.

##### KatA Proteolysis in Late Exponential Growth Phase Is Induced

As described before (19), and confirmed in this work (Fig. 1*A*), KatA polypeptide is not detectable in *E. faecalis* cell lysates from late stationary phase cultures lacking heme. The results shown in Fig. 1*B* suggested that apocatalase is degraded. When we added hemin to cells that were grown in heme-free medium until late exponential growth phase (OD_600_ = 1.7) catalase activity was detected already in the first sample taken after 15 min, and the addition of chloramphenicol prior to hemin did not inhibit formation of catalase (data not shown). This indicated simultaneous synthesis and degradation of apocatalase in late exponential growth phase cells.

**FIGURE 2.**
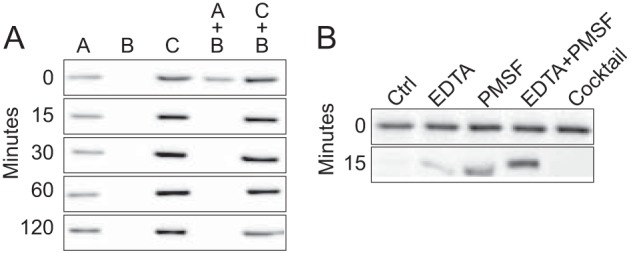
**Apocatalase polypeptide proteolysis is induced in late exponential growth phase *E. faecalis* cells.** *A*, lysates from strain OG1RF cells grown in heme-free medium until early exponential growth phase (*sample A*) or late exponential growth phase (*sample B*) and lysates from cells grown in heme-supplemented medium until late exponential growth phase (*sample C*) were analyzed individually or as mixtures as indicated. The samples were incubated at 37 °C, and the presence of KatA polypeptide in aliquots removed at the indicated time points was determined by immuno-blot. *B*, proteolysis of KatA in sample A mixed with sample B in the presence of 1 mm PMSF and/or EDTA or Complete EDTA-free protease inhibitor cocktail. The reference, sample A + B without any additions, is indicated by *Ctrl*.

To further investigate apocatalase proteolysis lysates from *E. faecalis* OGRF1 cells grown in heme-free medium until early and late exponential growth phase, respectively, were prepared and then mixed. The former lysate contained apocatalase, whereas the latter would contain protease activity. After incubation of the mixture for 15 min at 37 °C KatA polypeptide was no longer detectable by immuno-blot (Fig. 2*A*, *sample A* + *B*). When buffer was added instead of lysate from late exponential growth phase cells KatA was stable for at least 2 h at 37 °C (Fig. 2*A*, *sample A*). The addition of the serine protease inhibitor phenylmethylsulfonyl fluoride (PMSF) or EDTA resulted in protection of KatA from degradation in the mixture, and this was augmented when the compounds were combined (Fig. 2*B*). A commercial protease inhibitor cocktail (Complete mini EDTA-free, Roche Applied Science) did not protect KatA from degradation.

Holocatalase was not degraded in late exponential growth phase. To determine whether this was due to inhibition of the protease(s) by hemin we made use of the *katA*-deleted *E. faecalis* strain ELF7. A cell lysate of ELF7 grown in hemin-supplemented medium until late exponential growth phase was mixed with an OG1RF lysate containing apocatalase. Degradation of apocatalase occurred in this mixture. The combined results demonstrate that apocatalase is degraded by one (or several) proteases that are induced in late exponential growth phase and show that hemylation of KatA protects the protein from being degraded.

##### Purification of Apocatalase

The presence of KatA polypeptide in *E. faecalis* cells grown in heme-free medium offered the possibility to isolate preparative amounts of apocatalase. *E. faecalis* ELF7/pLUF15, a strain that lacks the chromosomal copy of *katA* and contains a multicopy plasmid encoding His_6_-tagged KatA, was grown in heme-free medium to overproduce apocatalase polypeptide. The cells were harvested from the culture at an OD_600_ ≤ 1.0 to avoid proteolytic degradation of KatA. Apocatalase was purified from the soluble cell fraction using affinity chromatography as described under “Experimental Procedures.” Holocatalase was in parallel purified from strain ELF7/pLUF15 grown in medium supplemented with 8 μm hemin.

**FIGURE 3.**
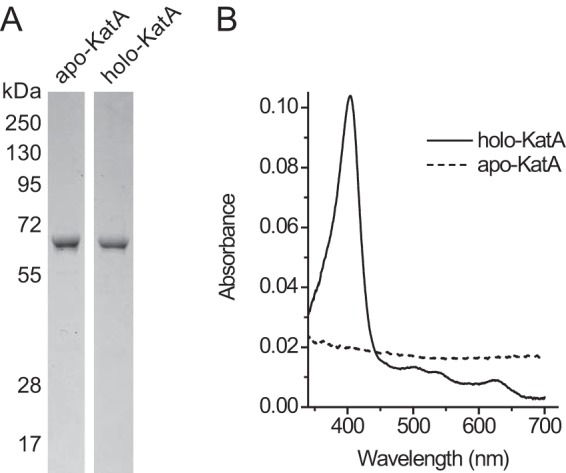
**Purity of preparations of apo- and holocatalase isolated from cytoplasmic extracts of *E. faecalis* strain ELF7/pLUF15 grown in heme-supplemented (holo-KatA) and heme-free (apo-KatA) medium.** *A*, Coomassie Brilliant Blue-stained SDS-PAGE gel. *B*, visible light absorbance spectra of corresponding amounts of purified apo- and holocatalase.

Preparations of purified apo- and holocatalase contained mainly KatA polypeptide as determined by SDS-PAGE (Fig. 3*A*) and confirmed by immuno-blot analysis using KatA antiserum and by mass spectrometry. The yield of apocatalase was about 0.25 mg/liter of culture. As expected isolated apocatalase lacked catalase activity (Table 2) and did not show the characteristic visible light absorbance spectral features of catalase with peaks at 407 (Soret), 530, and 630 nm (Fig. 3*B*).

**TABLE 2:** **Biochemical properties of isolated holo- and apocatalase and catalase assembled *in vitro* at two different temperatures** Data presented are the result from at least three independent preparations. Heme was determined by the pyridine hemochromogen procedure assuming 1 mol of protoheme IX per mol of KatA in isolated holocatalase. One unit (U) is 1 μmol of H_2_O_2_ degraded per min at the assay conditions used (see “Experimental procedures” for details). Numbers within parentheses show relative activities.

	Holocatalase	Apocatalase	*In vitro* assembled catalase	
			at 15 °C	at 37 °C
Protoheme IX/KatA (mol/mol)	1	<0.05	0.9 ± 0.1	0.8 ± 0.1
Catalase activity (U/pmol KatA)	4.2 ± 1.0 (100%)	<0.01 (<0.2%)	3.9 ± 2.0 (∼93%)	0.26 ± 0.05 (∼6%)

##### In Vitro Assembly of Catalase

**FIGURE 4.**
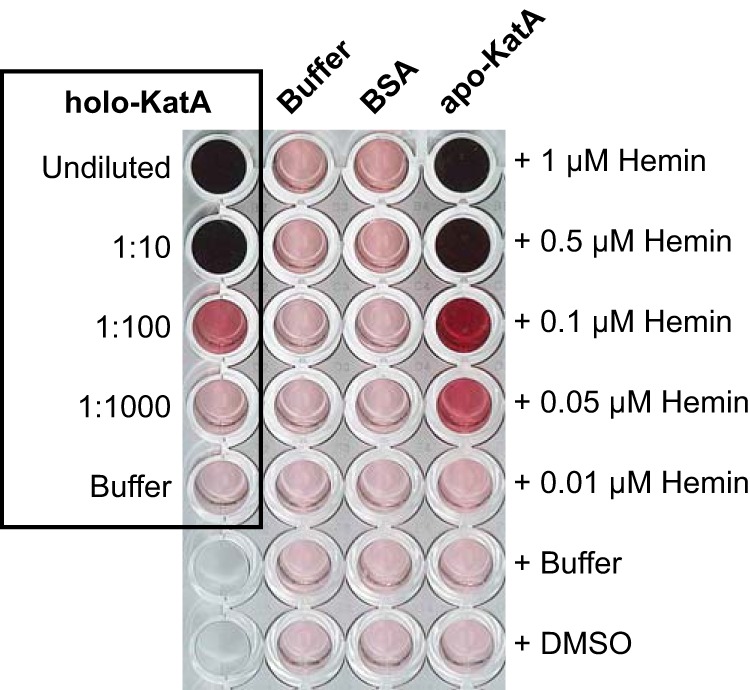
***In vitro* catalase assembly.** Purified apocatalase (1.5 μm KatA) or bovine serum albumin (BSA, 7.5 μm) in 50 mm potassium phosphate buffer, pH 8.0, or only buffer were mixed with hemin at different concentrations in microtiter plate wells. The final volume per well was 20 μl. The mixtures were incubated at 37 °C for 20 min. To each well was then added 200 μl of zymogram staining reagent, and catalase activity was registered by the development of purple color. Holocatalase (1.5 μm KatA) at different dilutions served as positive control.

Next we tested whether the purified apocatalase in the presence of heme can be assembled *in vitro* to form active enzyme. The first screens to find suitable experimental conditions for assembly were done in microtiter plates, and catalase activity was detected by zymogram staining. Incubation of hemin and apocatalase in phosphate buffer, pH 8.0, for 20 min at 37 °C resulted in catalase activity (Fig. 4). Control incubations with only apocatalase or hemin, or bovine serum albumin and hemin, yielded no detectable catalase activity.

**FIGURE 5.**
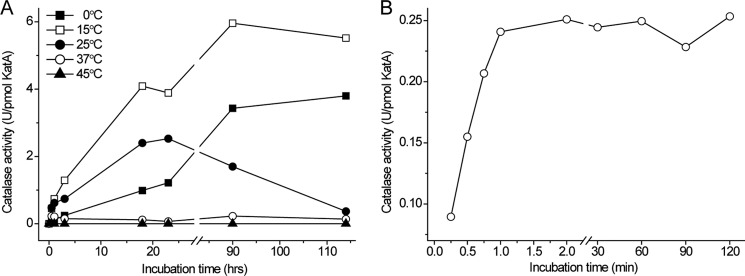
**Rate of *in vitro* catalase assembly and yield of catalase activity.** *A*, purified apocatalase (0.12 μm) was mixed with hemin (1.9 μm) in 50 mm phosphate buffer, pH 8.0, and then incubated at different temperatures. Catalase activity at 37 °C was determined on aliquots withdrawn from the incubation mixture at the indicated time points. *U*, units. *B*, apocatalase (0.5 μm) was mixed with 2 μm hemin and incubated at 37 °C in 50 mm phosphate buffer, pH 8.0. Catalase activity at 37 °C was determined at the indicated time points. The activity with only apocatalase was ≤0.01 units, and the activity of hemin in buffer at the corresponding concentrations was ≤0.02 units. Note the different time scale in *panels A* and *B*.

The rate at which catalase activity appears upon the addition of excess hemin to apocatalase was determined by using a spectrophotometer-based assay. This rate and the obtained final catalase activity were found to be strongly temperature-dependent (Fig. 5*A*). At 37 °C the activity increased promptly to reach a plateau of low activity within minutes (Fig. 5*B*). At 20 °C, or colder, the activity increased slowly, and it took many hours to reach the maximum level, which was more than 10-fold higher as compared with that obtained at 37 °C and similar to that of isolated holocatalase. Incubation of apocatalase with an excess of heme first at 37 °C for 2 h and then at 15 °C resulted in increased activity that finally reached a level similar to that of holocatalase. This demonstrated that the majority of apocatalase molecules in the presence of heme are stable at 37 °C and can mature into fully active enzyme when the temperature is lowered.

Apocatalase incubated with excess hemin at 15 and 37 °C, respectively, and then purified by affinity chromatography contained in both cases about one protoheme IX per KatA polypeptide (Table 2). The catalase that assembled at 15 °C showed ∼93% activity as compared with isolated *in vivo* assembled holocatalase. In contrast the assembly product obtained at 37 °C showed low (∼6%) catalase activity (Table 2). Supplementation of the buffer used for *in vitro* assembly with glycerol (30% w/v), NADPH (1 mm), Na-EDTA (2 mm); change of buffer to 20 mm MOPS; change of pH to 7.0; or variation in the amount (up to 5% v/v) of DMSO added with the hemin did not improve the yield of catalase activity resulting from incubation at 37 °C. The presence of imidazole (≥10 mm) and high concentrations of DMSO (≥5% v/v) decreased the yield of catalase activity.

**FIGURE 6.**
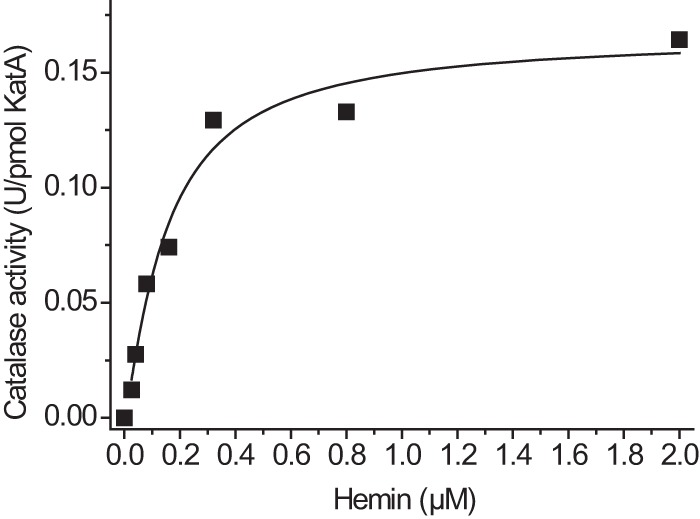
**Apocatalase heme affinity.** Aliquots of purified apocatalase (40 pmol of KatA) in 0.04–1 ml of 50 mm phosphate buffer, pH 8.0, were incubated with hemin (80 pmol) at concentrations ranging from 25 nm to 2 μm. Samples were incubated at 37 °C for 45 min, and after the addition of buffer to the final volume of 1 ml of catalase activity was determined at 37 °C. *U*, units.

The affinity of apocatalase for heme was analyzed by mixing 40 pmol of isolated apocatalase with 80 pmol of hemin. Hemin concentrations used ranged from 26 nm to 2 μm. Catalase activity was determined after incubation at 37 °C for 45 min. The experiment showed an apparent *K_D_* value of 150 nm (Fig. 6).

**FIGURE 7.**
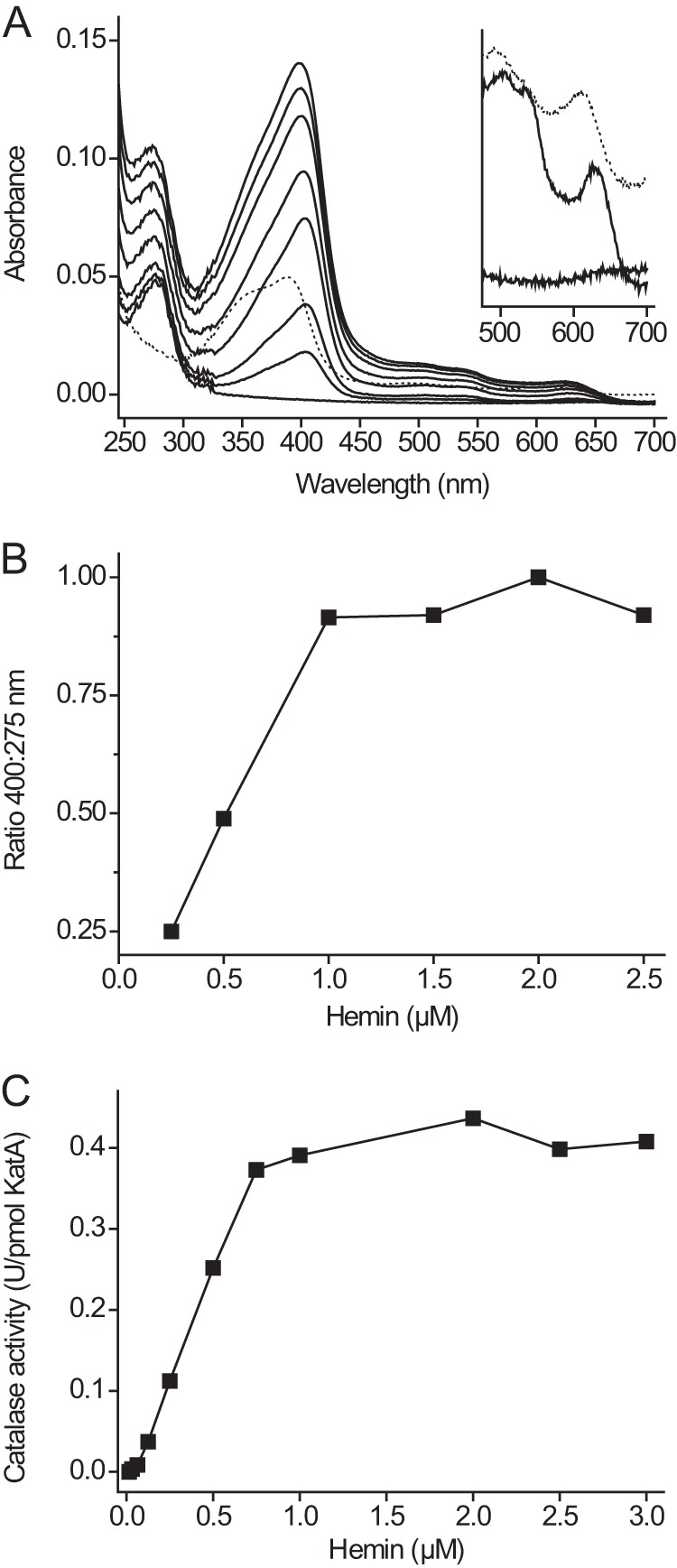
**KatA polypeptide to heme stoichiometry of *in vitro* hemylated catalase.** *A*, light absorption spectra of purified apocatalase (1.0 μm KatA) incubated at 20 °C with increasing concentrations of hemin (0.25–3 μm) in 50 mm phosphate buffer, pH 8.0. Spectra were recorded 2 min after the addition of hemin. The spectrum of 1 μm hemin is indicated as a *dotted trace*. The *inset* shows a magnification of the 500–700-nm spectral region for apocatalase, hemin, and hemylated KatA. *B*, the ratio of the Soret band peak (400 nm) to protein absorption peak (275 nm) of the spectra shown in *panel A* was calculated after subtraction of the background spectra from hemin. *C*, hemin at the indicated concentrations was added to purified apocatalase (1.0 μm KatA) in 50 mm phosphate buffer, pH 8.0. After 2 min at 20 °C the catalase activity at 37 °C was determined. *U*, units.

Fig. 7*A* shows light absorption spectra of 1 μm apocatalase to which increasing amounts of hemin have been added. The ratio of the Soret absorption peak and the 275 nm protein peak reached a maximum when KatA and hemin were present at approximately equimolar amounts (Fig. 7*B*). Catalase activity increased with the addition of hemin to reach a plateau at equimolar amounts of hemin and apocatalase (Fig. 7*C*). The visible light absorption spectrum of hemylated protein was similar to that of holocatalase (Fig. 3*B*). These results indicated near complete hemylation of apocatalase irrespective of the incubation temperatures used. This is consistent with the heme content of catalase assembled *in vitro* and then purified by affinity chromatography (Table 2). Apocatalase hemylated at 37 °C showed protease resistance, tested as before (Fig. 2) by incubation with an ELF7 extract from late exponential growth phase cells.

To judge the generality of the *in vitro* assembly system and exclude the possibility that the C-terminal hexahistidyl tag or the affinity chromatography system used influenced our results we repeated experiments with C-terminally StrepII-tagged apocatalase and holocatalase. These proteins were purified from *E. faecalis* strain ELF7/pLUMB38 (Table 1) as described under “Experimental Procedures.” The results were similar to those obtained with hexahistidyl-tagged KatA, demonstrating that the type of tag or affinity purification method used is not important for the *in vitro* assembly of catalase.

##### Hydrodynamic Properties of Apocatalase and Hemylated Catalase

**FIGURE 8.**
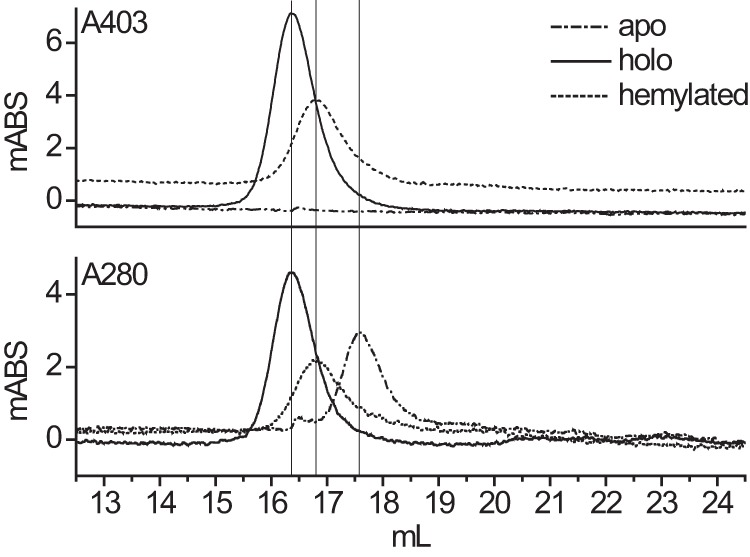
**Gel permeation chromatography elution profiles of purified holocatalase (*solid line*), *in vitro* hemylated catalase at 37 °C (*broken line*), and apocatalase (*dotted line*).** Absorption of the eluate was recorded at 280 and 403 nm to trace protein and the Soret band peak of catalase, respectively. *Vertical lines* indicate elution peak positions. The reference molecular size markers used, thyroglobulin (669 KDa) and bovine serum albumin (66 kDa), eluted at 13.4 and 17.2 ml, respectively (not shown). *mABS*, milliabsorbance.

To compare molecular properties of apocatalase and holocatalase as well as *in vitro* (at 15 and 37 °C) assembled catalase they were subjected to gel permeation chromatography. Holocatalase (tetramer) eluted at 16.4 ml, whereas apocatalase eluted at 17.6 ml (Fig. 8). At 37 °C the assembled catalase eluted at an intermediate position, 16.8 ml. The assembled catalase, which was fully active at 15 °C, eluted like holocatalase at 16.4 ml. The elution patterns as compared with those for thyroglobulin (669 kDa), catalase (216 kDa), and bovine serum albumin (66 kDa) suggested tentatively that apocatalase is monomeric.

The results confirm structural differences between the *in vitro* hemylated variant that assembled at 37 °C and holocatalase. The ratio of catalase activity to the amount of KatA antigen in fractions collected during the gel permeation chromatography showed that the low catalase activity observed in the catalase that assembled at 37 °C corresponded to a small amount of holocatalase, *i.e.* the enzyme activity peak eluted at 16.4 ml, whereas the majority of protein, registered as absorption at 280 and 410 nm, eluted at 16.8 ml. For holocatalase the elution profile of KatA and enzyme activity overlapped. The results suggest that most of the hemylated protein obtained in assembly at 37 °C is a temperature-trapped enzymatically defective intermediate structurally distinct from apo- and holocatalase.

##### Effect of a Pro-28 Mutation in KatA on Assembly of Catalase

The N-terminal arm of catalases contain conserved proline residues known to be crucial for the structure and assembly of the enzyme (2, 11). It was therefore of interest to analyze the biochemical properties of a previously isolated (17) *E. faecalis* KatA variant with a proline-28 to threonine substitution in the arm and compare properties of this protein with that of the temperature-trapped assembly intermediate.

*E. faecalis* strain EMB4 (Table 1) carries the *katA82* point mutation that causes the amino acid substitution at position 28 in KatA (17). Cell extracts of strain EMB4 grown overnight in heme-supplemented medium showed no KatA polypeptide nor catalase activity in the soluble (cytoplasmic) cell fraction. However, full-length KatA polypeptide, but no catalase activity, was found in the particulate (membrane) cell fraction. The KatA protein in the particulate fraction could be solubilized by 1% (w/v) SDS as well as by 8 m urea but not by non-ionic detergents, *i.e.* 1% (w/v) *n*-dodecyl-β-d-maltoside or 1% (w/v) Triton X-100 (immuno-blot not shown). These findings suggest that the mutant protein aggregates in the cell and for this reason co-fractionates with the particulate cell fraction.

For overproduction of P28T mutant KatA protein a plasmid containing the *katA82* gene (pLUMB31A) was constructed and introduced into *E. faecalis* EMB4. Strain EMB4/pLUMB31A grown in heme-free medium contained similar amounts of KatA polypeptide in the soluble and particulate cell fractions of samples taken during growth of the culture. At late exponential growth phase the mutant KatA was degraded in the soluble fraction but remained in the particulate fraction. Supplementation of the culture medium with 8 μm hemin increased the amount of soluble KatA but not that of aggregated KatA. However, after prolonged incubation (19 h to reach stationary phase) in the presence of hemin soluble KatA polypeptide was no longer detectable in strain EMB4/pLUMB31A, whereas aggregated KatA was still found. The results show that the P28T substitution in the N-terminal arm of KatA results in unstable protein that is prone to aggregation and unable to form active catalase but that might to some extent reversibly bind heme.

##### Cell Component(s) Promote Assembly of Catalase

Our finding of an *in vitro* assembly intermediate accumulating at 37 °C and slow formation of fully active catalase at lower temperatures appear incompatible with the *in vivo* results shown in Fig. 1 and a growth temperature of 37 °C for *E. faecalis* cells. One explanation for this contradiction could be that auxiliary cell components promote assembly of catalase. To analyze this we first prepared soluble cell fraction from *E. faecalis* ELF7/pLUF15 (overproduces KatA) and from strain ELF7 (lacks KatA), both grown to mid-exponential growth phase in heme-free medium. Heme at two concentrations (2 and 8 μm) was added to freshly prepared ELF7/pLUF15 soluble cell fraction. After incubation at 20 °C for 10 min KatA was immediately isolated by affinity chromatography. Catalase with near full activity (≥2.2 units/pmol KatA) was obtained at both hemin concentrations, showing that excess heme was available and that active catalase can be assembled rapidly in a cell-free extract.

**FIGURE 9.**
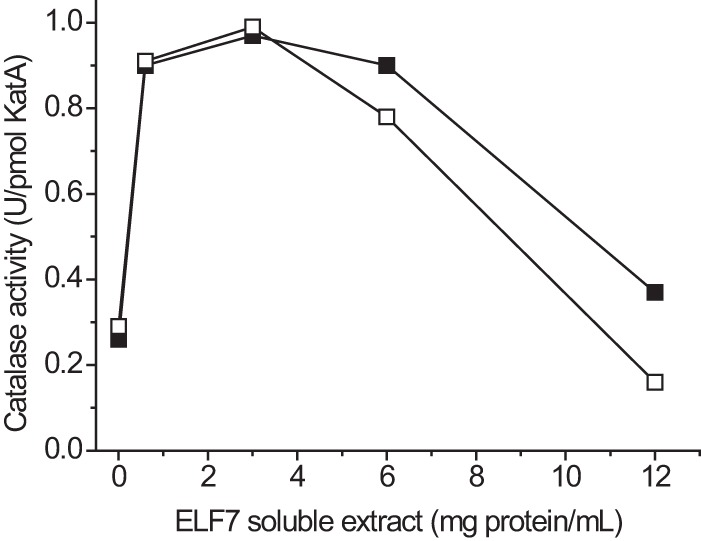
**Soluble cell factors promote maturation of catalase.** Apocatalase at 0.1 μm, 8 μm hemin, and different amounts of *E. faecalis* ELF7 soluble cell fraction (from bacteria grown in heme-free medium) were added to 50 mm potassium phosphate buffer, pH 8.0. After incubation for 10 min at 20 °C the mixtures were put on an ice water bath, and catalase activity was determined at 37 °C. *Filled symbols* indicate that the order of the addition of ingredients was extract, apocatalase, and finally hemin. *Open symbols* indicate that hemin was added before apocatalase. The presence of both hemin and apocatalase in the incubation mixture was required for formation of detectable catalase activity. *U*, units.

To confirm the presence of cell factors promoting assembly of catalase we added increasing amounts of *E. faecalis* strain ELF7 soluble cell fraction (lacks KatA polypeptide) to purified apocatalase and supplemented the mixtures with hemin. The soluble cell extract promoted formation of catalase activity (Fig. 9). These results indicate that one or more molecular chaperones or other types of auxiliary factors assist assembly of catalase in cells.

## DISCUSSION

Catalase has been under study for more than a century (20), but biogenesis of heme catalase is still not understood at the molecular level (9, 21). Formation of active catalase requires insertion of one heme group per subunit as well as assembly of the structurally intricate homo-tetramer. As determined from holoenzyme crystal structures, including that for *E. faecalis* catalase (10), the monomer in small subunit heme catalases is composed of four regions: the N-terminal arm (residues 4–50 in *E. faecalis*), the β-barrel (residues 51–300), the wrapping region (residues 301–415), and the C-terminal α-helical region (residues 416–477) (10). The heme pocket includes His-54, which is the active site heme distal essential histidine residue, the β-barrel, which by four strands forms most of the heme distal side, and the wrapping region with the heme proximal tyrosyl axial ligand (residue Tyr-337). In the assembled catalase the N-terminal arm of each monomer is intertwined with the wrapping region of another monomer.

In this work we have investigated expression of catalase in *E. faecalis* cells and have isolated catalase apoprotein and used it for *in vitro* assembly studies. *E. faecalis* cells cannot synthesize tetrapyrroles but grow well in the absence of heme and can take up heme from the environment to assemble heme proteins (7). We show that the *katA* gene is transcribed and translated independently of heme in the growth medium. Apocatalase was found to be degraded in cells by yet unidentified proteolytic activity induced at late exponential growth phase. Based on these findings we worked out a procedure for production and isolation of apocatalase: *E. faecalis* cells overproducing KatA with a C-terminal hexahistidyl or StrepII tag are grown in heme-free medium until mid-exponential growth phase, and apocatalase is purified as a monomeric protein from cell extract by affinity chromatography. Isolation of preparative amounts of apocatalase polypeptide from cells has to our knowledge only been reported once before: from a *Staphylococcus* auxotroph grown in the absence of heme (4).

Catalase was assembled *in vitro* by mixing purified apocatalase with hemin. At 15 °C this process was found to take many hours to be completed and yielded ≥90% active enzyme (as compared with holoenzyme assembled *in vivo*) (Table 2). Incubation at 37 °C, in contrast, resulted within minutes in hemylated enzymatically defective catalase. This form contained one protoheme IX group per KatA, showed a normal light absorption spectrum, was stable against proteolytic degradation, and differed in hydrodynamic property from both apocatalase and holocatalase as determined by gel permeation chromatography. These properties indicate that the heme pocket of catalase efficiently forms at 37 °C, but further maturation to active tetrameric enzyme is blocked at this temperature. Incubation of purified apocatalase with an excess of heme first at 37 °C followed by incubation at 15 °C for 24 h or longer yielded holocatalase. These results suggest that the form obtained at 37 °C is an assembly intermediate unable to finalize oligomerization. Because catalase inside *E. faecalis* cells rapidly assembles at 37 °C one or more molecular chaperones presumably facilitate maturation of catalase. We found evidence for the presence of such factors in soluble cell extracts (Fig. 9). Preparations of His- and StrepII-tagged apocatalase contained only one major protein, KatA (Fig. 3), and behaved similarly and reproducibly in the catalase assembly experiments. It can, however, not be excluded that our preparations of purified apocatalase contained small amounts of chaperones attached to a minority of the isolated apocatalase polypeptides, and this might explain the small fraction (<10%) of active catalase that rapidly assembled *in vitro* at 37 °C.

**FIGURE 10.**
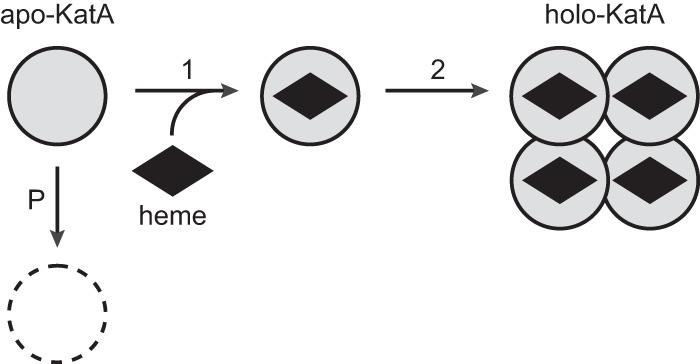
**Assembly of catalase from apoprotein and heme.** The schematic figure shows the order of events during biogenesis of catalase as suggested by our experimental results. *Step 1*, binding of heme to partially folded monomeric apocatalase. *Step 2*, oligomerization of hemylated subunits to form enzymatically active tetramer. This step is *in vitro* temperature-sensitive and rate-limiting. *Step P*, in *E. faecalis* cells in the absence of heme apocatalase is degraded by protease(s) induced at post-exponential growth phase.

The current model for catalase biogenesis, based on structural considerations and radioactive labeling studies with rat liver and yeast cells, proposes that monomer protein folding, heme insertion, and oligomerization are highly interconnected. Heme is thought to be inserted into apo-monomers generating a dimer that finally oligomerizes into the holo-tetramer (2, 11, 12). Our results from the use of purified components *in vitro* are in accordance with this model (Fig. 10). Formation of the heme pocket and incorporation of heme in the appropriate planar orientation apparently occur in concert because it involves three regions of the catalase polypeptide. Hemylation might induce protein dimerization as discussed in detail before (11). In the final homo-tetramer the heme group is hidden inside the protein and cannot be exchanged or reoriented. How the heme group is recognized by apocatalase (apparent *K_D_* of 150 nm; Fig. 6) and how it is delivered to apoprotein in the cell are not known. Initial contact between heme and polypeptide apparently does not include heme iron and the proximal tyrosine (Tyr-337) residue because *E. faecalis* catalase also assembles with metal-free protoporphyrin IX (19).

The N-terminal arm of heme catalase polypeptides contains two conserved proline residues (residues 28 and 49 in *E. faecalis* KatA). We found that a P28T mutation leads to aggregation of catalase polypeptide in the cell and that no catalytically active enzyme is formed. Heme to some extent promoted the solubility of the mutant protein variant in the cell, but in contrast to wild-type protein, did not protect it from degradation in the soluble cell fraction. This could indicate that interaction between the N-terminal arm and heme is an early reversible and essential step in the assembly pathway, but it could also merely reflect the importance of the conformation of the N-terminal arm in oligomerization of subunits (2).

We conclude that the methodology for production and purification of apocatalase presented in this work as well as our *in vitro* experimental results with apocatalase first reported here are valuable for research aimed at a molecular understanding of catalase biogenesis. The purification yield of apocatalase protein can in the future possibly be improved by construction of an *E. faecalis* strain lacking the protease(s) that degrade apocatalase in late exponential growth phase cells. The *in vitro* system offered here for assembly of catalase opens up new experimental approaches, for example, to identify molecular chaperones that assist in enzyme assembly and to study catalase oligomerization dynamically at the molecular level by various types of spectroscopy. Finally we wish to point out that the approach of using heme-less *E. faecalis* cells to produce apoprotein can most likely be applied on heterologous heme proteins. As one example we have produced the apo form of *E. coli* cytochrome *b*_562_.^3^

## Footnotes

3 M. Brugna, and L. Hederstedt, unpublished results.

## Footnotes

2 The abbreviations used are:

TSB: tryptic soy broth
TSBG: TSB supplemented with glucose
Bis-Tris: 2-(bis(2-hydroxyethyl)amino)-2-(hydroxymethyl)propane-1,3-diol
DMSO: dimethyl sulfoxide.
